# Combining Mutational Signatures, Clonal Fitness, and Drug Affinity to Define Drug-Specific Resistance Mutations in Cancer

**DOI:** 10.1016/j.chembiol.2018.07.013

**Published:** 2018-11-15

**Authors:** Teresa Kaserer, Julian Blagg

**Affiliations:** 1 Cancer Research UK Cancer Therapeutics Unit, The Institute of Cancer Research, London SM2 5NG, UK

**Keywords:** drug resistance, resistance hotspot, clonal fitness, mutation signature, targeted cancer drugs

## Abstract

The emergence of mutations that confer resistance to molecularly targeted therapeutics is dependent upon the effect of each mutation on drug affinity for the target protein, the clonal fitness of cells harboring the mutation, and the probability that each variant can be generated by DNA codon base mutation. We present a computational workflow that combines these three factors to identify mutations likely to arise upon drug treatment in a particular tumor type. The Osprey-based workflow is validated using a comprehensive dataset of ERK2 mutations and is applied to small-molecule drugs and/or therapeutic antibodies targeting KIT, EGFR, Abl, and ALK. We identify major clinically observed drug-resistant mutations for drug-target pairs and highlight the potential to prospectively identify probable drug resistance mutations.

## Highlights

• Drug affinity, clonal fitness, and mutation signatures define resistant mutations
• We report a computational work flow to identify drug-resistant hotspot mutations
• A comprehensive ERK2 resistance mutation dataset is used to validate the work flow
• Clinically relevant, drug-resistant mutations are identified

## Graphical Abstract

**Figure:**
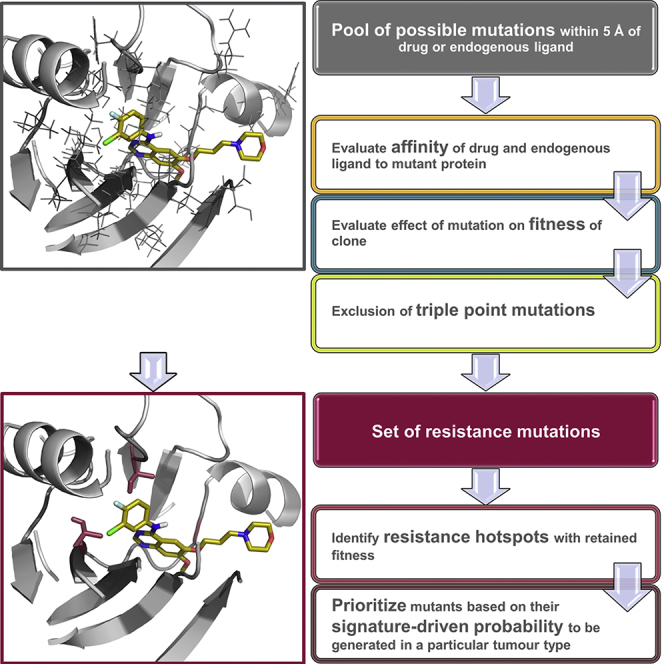

## Teaser

Resistance to targeted cancer drugs is a major challenge in therapy. Kaserer and Blagg report a computational approach to prospectively identify and prioritize clinically relevant drug-resistant mutations; it facilitates improved patient monitoring for the emergence of resistance and the timely discovery of durable treatment options.

## Introduction

Although targeted cancer therapies, for example, against kinases, hormone receptors, or hormone-synthesizing enzymes, have shown clinical success, many patients develop resistance to treatment and subsequently relapse. Second- and third-generation drugs are being developed to target these resistant mutants; however, there is a significant time span between the detection of clinically validated resistance mutations and the availability of suitably targeted treatment options. Early identification of drug-specific mutations is therefore critical and the aim of this study.

Several mechanisms underlying resistance to targeted drugs have been described (Holohan et al., 2013), including mutations directly affecting the drug target. Such mutations may, for example, increase affinity for the endogenous co-factor ATP, thereby decreasing the relative affinity of an ATP-competitive drug. Mutations within the binding site may also alter drug-protein interactions and directly interfere with drug binding (Barouch-Bentov and Sauer, 2011).

Several computational studies have investigated the impact of protein mutations on drug efficacy (Kamasani et al., 2017, Martínez-Jiménez et al., 2017, Paul et al., 2016, Reeve et al., 2015), mainly in an antiviral or antibacterial context (Frey et al., 2010, Hosseini et al., 2016, Fowler et al., 2018). However, rarely do these studies prospectively identify drug resistance mutations (Frey et al., 2010, Paul et al., 2016, Reeve et al., 2015). In predicting mutations that render *Staphylococcus aureus* resistant to an antifolate antibiotic, Reeve et al. (2015) evaluated the likely effect of possible mutations on both binding of the inhibitor and on binding of the endogenous ligand — an important aspect since any mutation that significantly abrogates the native activity of the wild-type (WT) protein is unlikely to survive selective evolutionary pressure (Gil and Rodriguez, 2016, Sprouffske et al., 2012, Pandurangan et al., 2017). However, Reeve et al. do not consider the likelihood of whether each mutation can be formed in bacteria.

In cancer, the mutation landscape of a tumor can be characterized by the mutational signatures operating in a particular cancer type (Alexandrov et al., 2013). These signatures describe the probability of a specific base exchange within a defined trinucleotide context. Some of these signatures have been associated with known mutagenic processes, such as UV irradiation or aging, while the mechanism of others still remains elusive (Alexandrov et al., 2013). These mutagenic processes can generate a single clone harboring the disease-causing “driver mutation,” which ultimately leads to the development of cancer (Greaves and Maley, 2012). In addition, non-transforming somatic mutations, so-called passenger mutations, are randomly created. While not oncogenic per se, passenger mutations can provide the substrate for an evolutionary advantage throughout cancer progression, for example, under the selective pressure of a targeted molecular therapy, leading to drug resistance. Known drug resistance mutations have not only been detected in treatment-naive patients (Inukai et al., 2006, Roche-Lestienne et al., 2002), but also in healthy individuals (Gurden et al., 2015). This suggests that small pools of viable treatment-resistant clones can pre-exist in patients and that drug treatment puts a selection pressure on a heterogeneous cancer cell population that selects for resistant sub-clones.

Each drug interacts with its biological target in a unique way, and each protein target mutation will differentially affect diverse classes of drugs. As a consequence, each compound can be expected to exhibit a unique resistance mutation profile. Three factors contribute to the probability and functional impact of a residue change: (1) the probability that the protein mutation can be generated from a DNA mutational signature (signature-driven probability), (2) whether the mutation maintains protein function and clones harboring the mutation are still viable (fitness), and (3) whether the mutation confers lower drug affinity with respect to the endogenous ligand for the target protein (affinity). Martínez-Jiménez et al. (2017) recently reported a workflow classifying potential drug resistance mutations based on Random Forest models and mutation signatures. However, the effect of mutations on the fitness of the clone was not taken into account. In addition, only single-point mutations (SPMs) were considered, despite the notable detection of double-point mutations (DPMs) in cancer patients (Table S1).

**Figure 1:**
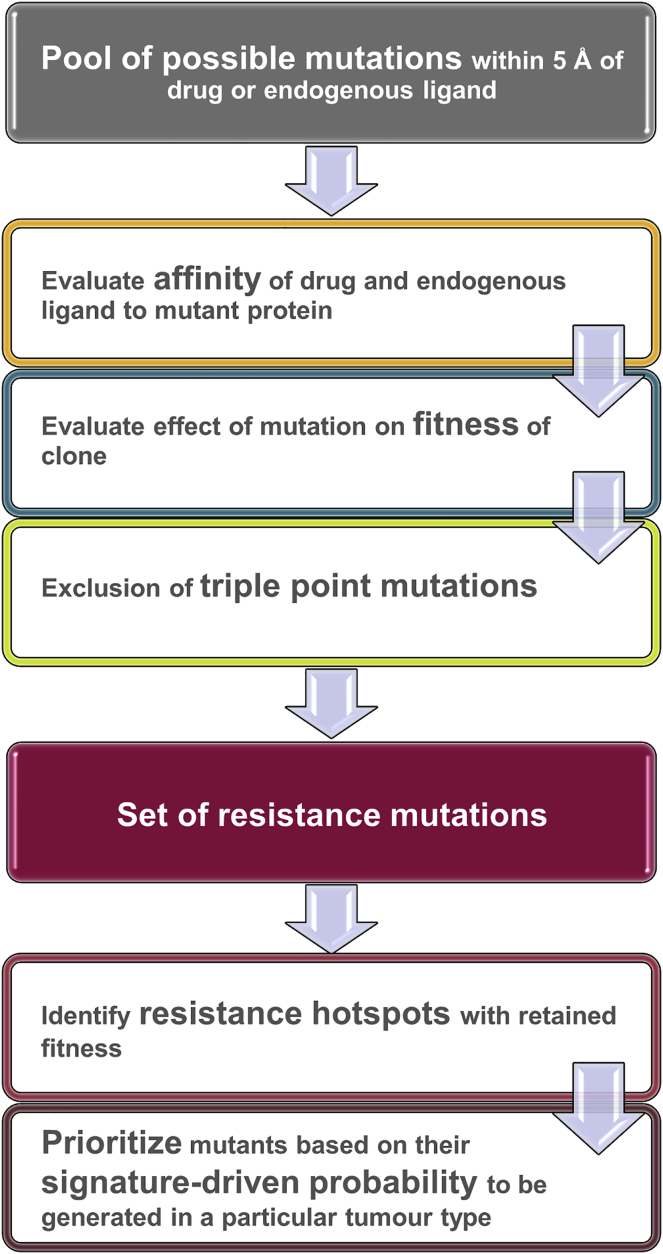
Workflow Potential mutations are evaluated based on their predicted effect on the affinity of both the drug and endogenous ligand (orange), the fitness of the resultant clone (blue), and the requirement for triple-point mutations to generate a mutant (lime green). Resistance hotspots are identified within the remaining set of resistant mutants; these resistance hotspots are protein residues where multiple amino acid changes are predicted to lead to resistance and which therefore have a high likelihood of functional relevance. Resistant mutations at these hotspots are prioritized based on the probability that they will be generated according to the known DNA mutational signatures operating in a particular cancer type.

We report an *in silico* cascade that sequentially evaluates the probability of generating any mutant within 5 Å of a bound ligand, the clonal fitness of each mutation, and the effect of each mutation on drug affinity in order to systematically and objectively prioritize mutations that are highly likely to arise under drug treatment. Importantly, our workflow classifies the impact of a mutation on drug affinity relative to endogenous ligand and does not rely upon accurate calculation of binding free energies; it also ranks mutations according to their likelihood of being generated in particular cancer type. The workflow (Figure 1 and described in detail below) is validated on a comprehensive benchmark dataset that describes the effect of nearly all possible extracellular signal-regulated kinase 2 (ERK2) missense mutations on sensitivity to the ERK2 inhibitor SCH772984 (Brenan et al., 2016). In addition, we apply the workflow to four well-established cancer targets and evaluate at least two US Food and Drug Administration-approved drugs (small molecules or biologics) per target.

## Results

### Affinity

In the first step, the software package Osprey (Chen et al., 2009, Gainza et al., 2013) was applied to evaluate the potential impact of protein active site mutations on both drug and endogenous ligand binding using protein crystallographic data.

Osprey uses ensembles of minimized side chain rotamers to calculate a K* score (Chen et al., 2009, Gainza et al., 2013), which estimates ligand binding to the evaluated protein construct. Importantly, by employing ensembles of rotamers, Osprey takes the dynamic nature of protein amino acid side chains into account.

K* scores were computed for the WT protein and all possible amino acid exchanges for both the drug and the endogenous ligand-protein complex with the exception of Pro, which cannot be processed by Osprey. We identify mutants that reduce affinity for the drug in comparison with the endogenous ligand and are therefore more likely to drive a viable resistant clone. Importantly, calculation of absolute drug affinity for WT and mutant proteins is not required. Please refer to the STAR Methods for additional details.

### Fitness

Clones harboring a mutant oncoprotein that is unable to bind its endogenous ligand lose their function and are unlikely to persist. All such deleterious mutants with a K* score of 0.0 for the endogenous ligand were discarded.

### Exclusion of Triple Point Mutations

Base codons for each WT residue were retrieved from the COSMIC database (Bamford et al., 2004). Amino acid exchanges that would require mutation of all three codon bases were discarded. We hypothesized that three contiguous coding base mutations are highly unlikely and, to the best of our knowledge, no such patient case has been reported. While the majority of drug resistance mutations arise from SPMs, we also included DPMs (so-called tandem mutations), which represent 5% (7 of 132) of unique drug resistance mutations with defined genetic alterations in the COSMIC database (access date 5 January 2018) and occur with similar frequency to insertions (Table S1).

### Resistance Hotspots and Signature-Driven Probability

Functional protein mutants were further triaged according to the number of predicted mutations at a specific residue position. This approach identifies resistance hotspots, where multiple different viable mutants are predicted to cause resistance to the drug under study. While comparing the prevalence of mutations at hotspots in a particular drug-target pair can be used for ranking positions, this method cannot be applied to cross-compare the prevalence of mutations at hotspots between drug-target pairs to infer global inter-target susceptibility to resistance. The top three ranked resistance hotspots were then analyzed in more detail in order to further prioritize amino acid changes at these positions that have a high probability (relP) of being generated in particular tumor types according to the DNA mutational signatures reported by Alexandrov et al. (2013). Each possible resistance hotspot mutation was ranked according to its derived relP and the top-ranked amino acid changes were considered most relevant. Please refer to the STAR Methods for additional details.

### ERK2: A Benchmarking Study

In 2016, Brenan and colleagues systematically studied the effect of a wide range of ERK2 genetic alterations on the cellular response to the ERK2 inhibitor SCH772984 (Brenan et al., 2016). They generated nearly every possible missense mutation using multiplexed site-directed mutagenesis. Pooled vectors were used to express ERK2 mutants in A375 human malignant melanoma cells; after drug exposure, the enrichment of resistant variants relative to their abundance in the initial cell population was determined by massively parallel sequencing.

This comprehensive dataset of possible resistant mutants to the ERK2 inhibitor SCH772984 provides a benchmark to evaluate the performance of our workflow. However, as a consequence of the aggressive mutant-generation protocol, this dataset includes protein mutants that require exchange of all three coding bases. Such mutants are excluded in our workflow (see above) and were also removed from the Brenan dataset.

**Figure 2:**
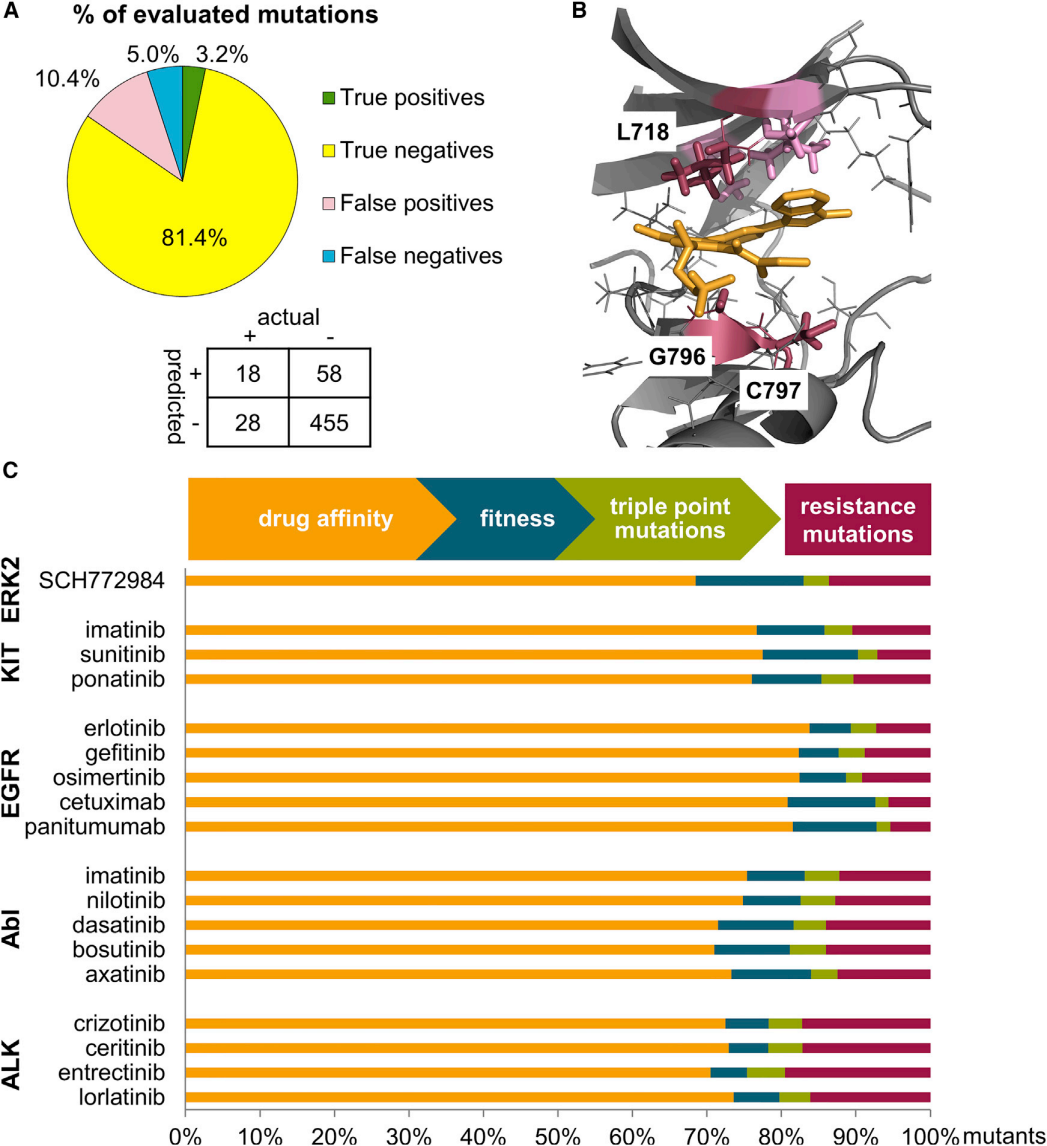
Predicted Resistance Mutations (A) Performance of the workflow on ERK2-SCH772984. The confusion matrix shows the absolute number of mutations (+, resistant; −, sensitive). (B) Predicted resistance hotspots that are consistent with clinically observed resistant mutants for the representative case study EGFR (gray) and osimertinib (orange), PDB: 4ZAU (Yosaatmadja et al., 2015). Residues within 5 Å of the ligand are depicted in gray, predicted and clinically observed resistance hotspots are highlighted as crimson sticks and labeled, predicted hotspot residues that have not yet been observed in the clinic are shown as pink sticks; figure created with PyMOL (PyMOL Molecular Graphics System, Version 1.7, Schrödinger, LLC). (C) Contribution of filtering steps to the identification of resistance mutations. The majority of mutants were discarded because they did not decrease drug affinity in comparison with binding of the endogenous ligand (orange). Mutations were further removed because of abrogated clonal fitness (blue) or because they required triple codon changes to be formed (green). The remaining pool of mutations (crimson) is predicted likely to confer resistance to drug treatment.

Brenan and colleagues reported 46 experimentally observed resistance mutations within 5 Å of the bound ligand that were not mutations to Pro or triple-point mutations. For SCH772984, 559 mutants covering 31 residues were evaluated by our workflow. The number of true-positive (TP) (mutants predicted to confer resistance that were confirmed by experimental testing), true-negative (mutants predicted to be sensitive that did not confer resistance in experimental testing), false-positive (FP) (mutants predicted to confer resistance that were found to be sensitive in experimental testing), and false-negative (mutants predicted to be sensitive, but conferred resistance in the experimental testing) predictions was calculated (Figure 2A).

Our workflow correctly classifies 84.6% of mutants and identifies almost 40% of the experimentally observed resistance mutations reported by Brenan et al. When considering only residue positions, 80% of experimentally observed mutated residues are discovered. The majority of experimentally evaluated ERK2 mutants remained sensitive to SCH772984 treatment and are correctly identified as sensitive by our approach, contributing to the high overall proportion (84.6%) of correct predictions. The low TP rate for SCH772984 (3.2% of mutants predicted to confer resistance were confirmed by experimental testing) is consistent with the experimental observation that only a small proportion of possible mutants (8.2%) cause drug resistance. The prioritized hotspot residues and their corresponding reported resistance mutations are provided in Table 1. For a detailed list of all predicted mutations please refer to Table S2.

**Table 1:** Prediction of ERK2 Mutations

Compound	No. of Pred Mut (No. of All Mut)^a^	Rank 1 Resistance Hotspot (No. of Mut)^b^	Experimentally Confirmed Resistance Mutants at Hotspot Residue^c^	Rank 2 Resistance Hotspot (No. of Mut)^b^	Experimentally Confirmed Resistance Mutants at Hotspot Residue^c^	Rank 3 Resistance Hotspot (No. of Mut)^b^	Experimentally Confirmed Resistance Mutants at Hotspot Residue^c^
SCH772984	76 (559)	Y64 (10)	Y64I^d^	Y36 (9)	Y36R^d^	NA^e^	NA
			Y64L^d^	D111 (9)	Y36N^d^		
			Y64V^d^		Y36Q^d^		
					Y36G^d^		
					Y36I^d^		
					Y36L^d^		
					Y36V^d^		

See also Table S2.

a The number of mutants predicted to confer resistance (no. of pred mut) from the initial pool of possible mutants within 5 Å of the ligand (no. of all mut).

b Resistance hotspots are identified and ranked according to the number of viable mutants (no. of mut) predicted for that residue.

c Experimentally observed resistance mutations are highlighted for each resistance hotspot.

d Mutants of resistance hotspots were not further ranked based on their relP in this case because Brenan et al. evaluated the mutants in cell lines and the clinical relevance of the mutants for the different cancer types is not known.

e NA, not applicable, tied resistance hotspot at rank 2.

### Clinical Case Studies

We studied four protein kinases (KIT, epidermal growth factor receptor [EGFR], breakpoint cluster region-Abelson [Bcr-Abl] kinase, and anaplastic lymphoma kinase [ALK]). In each case protein-ligand crystal structures of approved drugs exemplify multiple generations of compound design; furthermore, as inhibitors of these targets provide the current standard of care for multiple cancer types, clinically observed resistance mutations are documented for most compounds and provide the potential for clinical validation of our computational workflow.

### KIT

The first-generation inhibitor imatinib is resistant to the KIT gatekeeper mutation T670I (Antonescu et al., 2005, Tamborini et al., 2004). We investigated 648 possible mutations, all of which lie within 5 Å of either imatinib or ADP; 68 mutations remained after filtering for reduced drug binding, fitness of the clones, and triple-point mutants. For residue T670, eight mutations are predicted to cause resistance to imatinib, making it the first-ranked resistance hotspot; within this set, gatekeeper mutation T670I has the highest relP. Furthermore, the known resistance mutation T670E (Wardelmann et al., 2005) is ranked sixth by relP. Positions C809 and V668 are identified as second- and third-ranked resistance hotspots, respectively. C809R, the mutation with the highest relP, has been reported in a patient diagnosed with myelodysplastic syndrome-derived leukemia (Lorenzo et al., 2006), highlighting its biological relevance; however, similar to all predicted V668 mutations, this residue has not yet been associated with imatinib resistance.

The second-generation inhibitor sunitinib is reported to overcome resistance to the gatekeeper mutation T670I and, consistent with these reports, T670I/E mutations are not predicted to elicit sunitinib resistance by our workflow. Indeed, none of our predicted resistance mutations to sunitinib has yet been associated with clinically observed resistance. Only one resistance mutation has so far been reported for sunitinib in the COSMIC database (Bamford et al., 2004, access date 23 November 2017); this mutation is not within the ATP-binding site.

Ponatinib has been shown to bind KIT, but is not approved for KIT-associated cancer types and clinical data are therefore lacking. Our method predicts resistance to ponatinib due to less-favorable interactions with the gatekeeper T607I mutation; however, *in vitro* data suggest that this mutant is sensitive to ponatinib treatment (Garner et al., 2014). Prioritized KIT resistance hotspot residues and their corresponding reported clinical resistance mutations are provided in Table 2. A full list of predicted resistance mutations and their relP values is available in Table S3.

**Table 2:** Prioritized KIT Resistance Mutations

Compound	No. of Pred Mut (No. of All Mut)^a^	Rank 1 Resistance Hotspot (No. of Mut)^b^	Confirmed Clinical Resistance Mutants (Rank relP)^c^	Rank 2 Resistance Hotspot (No. of Mut)^b^	Confirmed Clinical Resistance Mutants (Rank relP)	Rank 3 Resistance Hotspot (No. of Mut)^b^	Confirmed Clinical Resistance Mutants (Rank relP)
Imatinib	68 (648)	T670 (8)	T670I (1) (Antonescu et al., 2005, Tamborini et al., 2004) T670E (6) (Wardelmann et al., 2005)	C809 (7)	NR^d^	V668 (6)	NR
Sunitinib	33 (468)	A814 (6)	NR	G596 (5)	NR	A621 (4) V654 (4)	NR
Ponatinib	64 (630)	C809 (8)	NR	T670 (5) C788 (5)	(T670I)^e^	NA^f^	–

See also Table S3.

a Number of mutants that have been predicted to confer resistance (no. of pred mut) from the initial pool of possible mutants within 5 Å of the ligand (no. of all mut).

b Resistance hotspots are identified and ranked according to the number of viable mutants (no. of mut) predicted for a residue.

c RelP was calculated for all resistance hotspot mutations. Clinically observed resistance mutations and their rank according to relP (rank relP) are highlighted for each resistance hotspot.

d NR, not reported––none of the predicted mutations has yet been reported to confer resistance to the drug.

e T670I was predicted to confer resistance to ponatinib; however, T670I is reported to be sensitive to ponatinib (Garner et al., 2014).

f NA, not applicable, tied resistance hotspot at rank 2. The gatekeeper mutation is underlined.

### EGFR

Applying our workflow to the first-generation EGFR inhibitor erlotinib revealed that G796 is the resistance hotspot with the most predicted mutations. Consistent with this prediction, the G796R mutation with the second-ranked relP, has been reported to weaken the affinity for erlotinib in comparison with the WT enzyme (Avizienyte et al., 2008). T790 is the second-ranked resistance hotspot with gatekeeper mutation T790M (Pao et al., 2005) having the second highest relP. L718, L788, and T854 share the third-ranked hotspot, with three predicted mutations each. L788F has been detected in lung adenocarcinoma patient samples highlighting its clinical relevance (Liu et al., 2013); however, similar to all other third-rank mutations, L788F has not yet been associated with clinical resistance to erlotinib. Gefitinib, another first-generation EGFR inhibitor, elicits a similar predicted resistant mutation profile to erlotinib (Table 3).

**Table 3:** Prioritized EGFR Resistance Mutations

Compound	No. of Pred Mut (No. of All Mut)^a^	Rank 1 Resistance Hotspot (No. of Mut)^b^	Confirmed Clinical Resistance Mutants (Rank relP)^c^	Rank 2 Resistance Hotspot (No. of Mut)^b^	Confirmed Clinical Resistance Mutants (Rank relP)	Rank 3 Resistance Hotspot (No. of Mut)^b^	Confirmed Clinical Resistance Mutants (Rank relP)
Erlotinib	34 (469)	G796 (9)	NR^d^	T790 (5)	T790M (2) (Pao et al., 2005)	L718 (3) L788 (3) T854 (3)	NR
Gefitinib	43 (487)	G796 (14)	G796A (3) (Uramoto et al., 2007)	T790 (6)	T790M (2) (Kobayashi et al., 2005, Pao et al., 2005)	T854 (4)	NR
Osimertinib	38 (415)	G796 (14)	G796S (1) (Ou et al., 2017b) G796D (2) (Zheng et al., 2017) G796R (5) (Ou et al., 2017b)	C797 (4)	C797R (3) (Ou et al., 2017b)	L718 (3) G719 (3) V726 (3) A743 (3)	NR
Cetuximab	65 (1,153)	G471 (15)	NR	G441 (12)	G441R (1) (Braig et al., 2015)	S418 (10)	NR
Panitumumab	65 (1,207)	S418 (14)	NR	G441 (12)	G441R (1) (Braig et al., 2015)	G471 (8)	NR

See also Table S4.

The gatekeeper mutation is underlined.

a The number of mutants predicted to confer resistance (no. of pred mut) from the initial pool of possible mutants within 5 Å of the ligands (no. of all mut).

b Resistance hotspots are identified and ranked according to the number of viable mutants (no. of mut) predicted for a residue.

c The relP was calculated for all resistance hotspot mutations. Clinically observed resistance mutations and their rank according to relP (rank relP) are highlighted for each resistance hotspot.

d NR, not reported––none of the predicted mutations are reported to confer resistance against the drug.

The third-generation inhibitor osimertinib binds reversibly to EGFR prior to covalent bond formation (Yosaatmadja et al., 2015). Osimertinib was reported to overcome resistance to T790M and, consistent with this finding, the T790M mutation is not predicted to elicit resistance to osimertinib according to our protocol. Residues G796 (14 mutations), C797 (4 mutations), L718, G719, V726, and A743 (3 mutations each) are the top-ranked hotspot residues predicted to confer resistance. During the preparation of this manuscript, osimertinib resistance mutations affecting C797 (Ou et al., 2017b), G796 (Ou et al., 2017b, Zheng et al., 2017), and L718 (Ou et al., 2017a) (Figure 2B) were reported in clinical studies, building confidence in the prospective utility of our approach.

Further to small-molecule EGFR inhibitors, anti-EGFR monoclonal antibodies cetuximab and panitumumab are approved for the treatment of metastatic colorectal cancer. In contrast to small-molecule ATP-competitive kinase inhibitors, cetuximab and panitumumab interact with the extracellular domain of EGFR.

We evaluated the effect of extracellular domain mutations on antibody affinity and affinity of the endogenous ligands EGF and transforming growth factor α (TGF-α)––interactions that have been characterized by protein structural data. Both EGF and TGF-α are peptidic macromolecules with larger interaction surfaces compared with small molecules; thus evaluation of a higher number of possible mutations was necessary to ensure coverage of the extensive protein-protein interaction interface (1,153 and 1,207 mutations for cetuximab and panitumumab, respectively).

For both cetuximab and panitumumab, resistance mutation G441R (residue G465R in the mature protein sequence including the signaling peptide [mps]) has been detected in the clinic (Braig et al., 2015). G441 is the second-ranked resistance hotspot, with mutation G441R ranked first according to relP for both antibodies (Table 3). Confirmed clinical resistance mutations to cetuximab have also been identified at residues S468 (S468R [S492R in the mps]) (Montagut et al., 2012) and I467 (Arena et al., 2015) (I467M [I491M in the mps]); the fourth- and fifth-ranked resistance hotspots in our workflow (Table S4).

Our top-ranked predicted hotspot residues, S418 and G471 have not yet been associated with clinical resistance to cetuximab or panitumumab; however, seven endogenous ligands can activate EGFR (Schneider and Wolf, 2009) of which only EGF and TGF-α interactions are characterized by protein structural data. Residues S418 and G471, while not predicted to affect the interaction of EGFR with endogenous ligands EGF and TGF-α, may be important for interaction with one or more of the remaining five endogenous ligands such that mutation at these residues reduces clonal fitness and persistence. For example, we predict that several mutations affecting EGFR residues S440 and V417 tolerate TGF-α binding, but abrogate EGF binding, and are therefore filtered out in our workflow. Given the lack of structural data for the majority of endogenous EGFR ligands, and the number of potential mutations, the identification of clinically relevant resistance mutations is encouraging. All prioritized EGFR hotspot residues and corresponding reported clinical resistance mutations are summarized in Table 3. A list of all predicted mutations and relPs is provided in Table S4.

### Abl

The hotspot residue with the highest number of predicted mutants for the Abl inhibitor imatinib is A380, for which clinically observed occurrences have yet to be reported. The second-rank predicted hotspot is shared by residues V256, A269, and T315. For V256 and T315, resistance mutations V256L (Bamford et al., 2004, Tiribelli et al., 2013), gatekeeper mutation T315I (Gorre et al., 2001), and T315V (Redaelli et al., 2012) have all been identified in the clinic (Table 4). The contribution of mutational signatures to the landscape of mutations has not yet been reported for chronic myeloid leukemia (CML), and therefore the relP for hotspot mutations predicted for Abl residues could not be calculated.

**Table 4:** Prioritized Abl Resistance Mutations

Compound	No. of Pred Mut (No. of All Mut)^a^	Rank 1 Resistance Hotspot (No. of Mut)^b^	Confirmed Clinical Resistance Mutations^c^	Rank 2 Resistance Hotspot (No. of Mut)^b^	Confirmed Clinical Resistance Mutations	Rank 3 Resistance Hotspot (No. of Mut)^b^	Confirmed Clinical Resistance Mutations
Imatinib	66 (540)	A380 (8)	NR^d^	V256 (7) A269 (7) T315 (7)	V256L (Bamford et al., 2004, Tiribelli et al., 2013) T315I (Gorre et al., 2001)	NA^e^	–
Nilotinib	69 (540)	V256 (8) A380 (8)	NR	NA	–	Y253 (6) A269 (6) T315 (6) G321 (6)	T315I (Bamford et al., 2004, Redaelli et al., 2012, Weisberg et al., 2005)
Dasatinib	58 (414)	A380 (12)	NR	L248 (7) A269 (7)	NR	NA	–
Bosutinib	58 (414)	V299 (7) T315 (7) G321 (7)	V299L (Jabbour et al., 2012, Redaelli et al., 2012) T315I (Cortes et al., 2011, Redaelli et al., 2012)	NA	–	NA	–
Axitinib	52 (396)	V256 (8)	NR	G321 (7)	NR	L248 (6) A269 (6) A380 (6)	NR

See also Table S5.

a The number of mutants predicted to confer resistance (no. of pred mut) from the initial pool of possible mutants within 5 Å of the ligands (no. of all mut).

b Resistance hotspots are identified and ranked according to the number of viable mutants (no. of mut) predicted for a residue.

c Clinically observed resistance mutations are highlighted for each resistance hotspot. The relP could not be calculated as signatures for CML were not available.

d NR, not reported––none of the predicted mutations were reported to confer resistance. In the case of axitinib, clinical resistance data on Abl are not yet available.

e NA, not applicable, tied resistance hotspot at rank 1 or 2. The gatekeeper mutation is underlined.

We obtained similar and consistent predictions for nilotinib, dasatinib, and bosutinib (Table 4). Additional resistance mutations affecting L248 are predicted for dasatinib consistent with *in vitro* data (Redaelli et al., 2012). While these mutations have been observed in the clinic (Redaelli et al., 2012), they have not yet been reported as resistant mutants for patients treated with dasatinib. Similarly, the effect of the predicted V256L resistant mutation on nilotinib activity has not been reported.

Axitinib has been reported to overcome resistance to the T315I gatekeeper mutation of Abl (Pemovska et al., 2015). Although not ranked among the top three resistance hotspots, axitinib is predicted to be resistant to T315I. Crystallographic studies of the T315I mutation in complex with axitinib revealed that this mutation causes large conformational changes of Abl compared with WT protein (Pemovska et al., 2015), which may not be adequately captured by our method. The L248R mutation, reported to confer resistance to axatinib *in vitro* (Pemovska et al., 2015), is prioritized by our workflow; however, clinical resistance data for axitinib targeting Abl is not yet available. A summary of the prioritized Abl hotspot residues and their corresponding reported clinical resistance mutations are provided in Table 4. A full list of predicted resistance mutations is provided in Table S5.

### ALK

The hotspot residue with the highest frequency of predicted mutations for the first-generation ALK inhibitor crizotinib is G1269 (13 mutants). The clinically observed resistance mutation G1269A (Doebele et al., 2012, Gainor et al., 2016) and G1269S and G1269C mutations (Zhang et al., 2011), which confer resistance *in vitro*, rank at positions four, seven, and eight, respectively, based upon their relP values. For the second-ranked resistance hotspot G1202, 12 mutants were predicted and the known resistance mutation G1202R (Gainor et al., 2016, Katayama et al., 2012) has the second highest relP. Hotspots I1122, G1201, and D1203 rank third equal. While no G1201 mutations are associated with resistance, the D1203N mutation confers resistance *in vitro* (Gainor et al., 2016, Heuckmann et al., 2011) and has been associated with resistance in patients (Zhang et al., 2016). I1122V has been detected in a resistance screen against the second-generation inhibitor brigatinib, and has also been confirmed to confer resistance to crizotinib (Ceccon et al., 2015). We generated similar results for the second-generation inhibitor ceritinib (Table 5), although, in this case, the G1269A mutation was not predicted to cause resistance, consistent with *in vitro* and clinical data (Gainor et al., 2016, Shaw et al., 2015).

**Table 5:** Prioritized ALK Resistance Mutations

Compound	No. of Pred Mut (No. of All Mut)^a^	Rank 1 Resistance Hotspot (No. of Mut)^b^	Confirmed Clinical Resistance Mutants (Rank relP)^c^	Rank 2 Resistance Hotspot (No. of Mut)^b^	Confirmed Clinical Resistance Mutants (Rank relP)	Rank 3 Resistance Hotspot (No. of Mut)^b^	Confirmed Clinical Resistance Mutants (Rank relP)
Crizotinib	65 (378)	G1269 (13)	G1269A (4) (Doebele et al., 2012, Gainor et al., 2016)	G1202 (12)	G1202R (2) (Gainor et al., 2016)	I1122 (7) G1201 (7) D1203 (7)	NR^d^
Ceritinib	71 (414)	G1202 (12)	G1202R (2) (Gainor et al., 2016)	G1269 (10) D1203 (10)	D1203N (1) (Gainor et al., 2016)	NA^e^	–
Entrectinib	88 (451)	G1123 (15)	NR	G1269 (13)	NR	G1202 (12)	NR
Lorlatinib	58 (360)	G1269 (13)	NR	G1123 (8) D1203 (8)	NR	NA	–

See also Table S6.

a Number of mutants that have been predicted to confer resistance (no. of pred mut) from the initial pool of possible mutants within 5 Å of the ligand (no. of all mut).

b Resistance hotspots are identified and ranked according to the number of viable mutants (no. of mut) predicted for each residue.

c RelP was calculated for all resistance hotspot mutations. Clinically observed resistance mutations and their rank according to relP (rank relP) are highlighted for each resistance hotspot.

d NR, not reported––none of the predicted mutations were reported to confer resistance against the drug. In the case of entrectinib and lorlatinib clinical resistance data are not yet available.

e NA, not applicable, tied resistance hotspot at rank 1 or 2.

Entrectinib and lorlatinib are investigational ALK inhibitors currently in clinical trials. Increased half maximal inhibitory concentration values (decreased binding) in comparison with the WT protein have been demonstrated in *in vitro* experiments for several of the mutants prioritized by our workflow. In particular, G1269A (Ardini et al., 2016) and G1202R (Ardini et al., 2016) for entrectinib and G1269A (Gainor et al., 2016, Johnson et al., 2014, Shaw et al., 2015) and D1203N (Gainor et al., 2016) for lorlatinib. In addition, the G1123S and G1123D mutants were identified in an *in vitro* resistance screen for the ALK inhibitor TAE684 (Heuckmann et al., 2011) and in a patient resistant to ceritinib (Toyokawa et al., 2015). However, no data are currently available on entrectinib. The prioritized hotspot residues for ALK and corresponding reported clinical resistance mutations are provided in Table 5. A comprehensive list of all predicted resistance mutations is provided in Table S6.

## Discussion

We present a computational workflow to identify clinically relevant drug resistance mutations to targeted cancer therapies, both small molecule and biological. The workflow consists of consecutive filtering steps addressing three factors, which determine whether a mutation is likely to confer resistance in the clinic. Of these, the activity cutoff for protein-drug affinity compared with affinity for the endogenous ligand proved the most important and excluded 75.8% ± 1.1% (n = 18 [all 18 target-interaction partner case studies] ±SEM) of all potential mutants for each drug-target combination (Figure 2C). This filtering step contains a fitness component; all mutants with predicted decreased affinity for the endogenous ligand (i.e., less fit clones), but with equivalent or increased affinity for the drug (i.e., more sensitive to drug treatment) are not progressed. In further considering the potential for disrupted binding of an endogenous ligand to mutated proteins, 8.6% ± 0.7% (n = 18, ±SEM) of mutants are predicted to completely abrogate binding of the endogenous ligand and were discarded. Removing triple-point mutations excluded a further 3.7% ± 0.2% (n = 18, ±SEM) of possible mutants. Interestingly, the majority of remaining mutations predicted to cause resistance (68.1% ± 1.7% [n = 18, ±SEM]) arise from DPM consistent with a similar proportion of DPMs observed in the Brenan dataset (Brenan et al., 2016). While DPMs commonly exhibit a low relP, they may become relevant when the predominant resistance clone harboring an SPM has been eradicated by cancer therapy. Taken together, 11.8% ± 1.0% (n = 18, ±SEM) of all evaluated mutations were considered to cause resistance (Figure 2C).

The pool of potential resistant mutations was further analyzed to identify resistance hotspots where multiple different viable mutants are predicted to cause resistance to the drug under study. Mutants at these hotspots were prioritized based on their relP, which quantifies the relative probability of each specific amino acid mutation in the context of a defined cancer type. Applying this workflow, we correctly classify 84.5% of mutations in a comprehensive ERK2 mutation dataset and identify almost 40% of mutants conferring resistance to the ERK2 inhibitor SCH772984. Furthermore, we identify clinically observed drug resistance mutations within the top three predicted hotspot residues for first-generation compounds in all cases studied and for most second- and third-generation compounds where clinically observed resistance mutations have been reported. Importantly, in all cases studied, except ALK, the gatekeeper mutation is predicted among the top three resistance hotspots. In the ALK case study, two mutations that are also commonly observed in the clinic are highly ranked by our method. For the osimertinib-EGFR drug-target pair, we predicted resistance mutations before they were confirmed by clinical reports.

Our workflow highlights mutations not yet observed in the clinic, or that may constitute FP predictions. The number of mutants progressed to further evaluation can be user defined. In the cases exemplified here, we prioritize three mutations with the highest relP for the top three resistance hotspots and significantly narrow the pool of potential resistance mutations for each drug from ∼350–1,200 possibilities to 9. While it may not be practical to further explore many hundreds of possible mutations, we suggest that a set of 9 prioritized mutants is more amenable to experimental testing within a drug discovery project or clinical setting. Thus, this workflow facilitates more focused monitoring of potential resistant mutations, as well as the design of next-generation compounds sensitive to likely resistant mutants.

Notably, our workflow evaluates the effect of a mutation on drug binding with respect to the WT protein. In some cases, exquisite drug affinity for the WT protein may mitigate a mutation-driven loss of potency. For example, our workflow correctly predicted the reduced affinity of lorlatinib for ALK G1269A; however, the exquisite potency of lorlatinib for WT ALK may render the relative loss of affinity for ALK mutants inconsequential (Gainor et al., 2016, Johnson et al., 2014, Shaw et al., 2015).

The workflow is dependent upon the availability and quality of both protein-ligand structural information and mutational signatures across diverse tumor types. For example, it is not clear which mutational signatures operate in CML, and, as a consequence, we could not determine the relP for mutants predicted to interfere with binding of Abl inhibitors in CML.

The approach presented here investigates the effects of protein mutation on drug binding mode as characterized by the input protein-ligand structure. How such mutations affect the overall conformation of the target protein and/or drug-target complex is not encompassed by our method. Extension of the approach by considering the effect of residue mutation on global protein conformation and by inclusion of structural models for highly homologous protein families would further expand the potential impact and is the aim of our future studies. For example, FP mutations that stabilize inactive protein conformations that are unlikely to persist could be excluded, and mutations that further stabilize an active protein conformation could be included. Furthermore, drug-resistant mutations distant from the binding site, including those which influence protein flexibility and conformation, which are beyond the scope of our current method, could also be evaluated.

This workflow includes three critical determinants of clinically relevant drug-resistant mutations. Importantly, the workflow can be further adapted to test more specific hypotheses. For example, Osprey parameters can be changed to include larger backbone movements and/or to include multiple ligand rotamers; furthermore, the number of prioritized mutants can also be user-defined.

In conclusion, we have developed and validated a computational method to prospectively identify clinically relevant drug-resistance mutations. We suggest that this approach can have a significant impact on the design and development of targeted therapies by proactively signposting drug resistance hotspots. Prior knowledge of resistant mutants enables their timely detection in patients and the early development of effective treatment options against the resistant tumor cell population.

## Significance

**Although molecularly targeted cancer therapies have shown great success in the clinic, drug resistance has emerged as the major challenge. Resistance mutations are commonly identified and characterized during clinical evaluation, often resulting in a reactive approach to tackling drug resistance. We report a computational method to prospectively identify drug resistance mutations during the design phase of potential therapeutics. This approach enables early signposting of likely resistance hotspots and supports more focused monitoring of potential emergent resistant clones as well as the timely development of alternative treatment options.**

## STAR★Methods

### Key Resources Table

**Table:** 

REAGENT or RESOURCE	SOURCE	IDENTIFIER
**Deposited Data**		

Crystal structures of target-ligand complexes	The Protein Data Bank	Table S7; http://www.rcsb.org/pdb/home/home.do
Mutation signatures	Alexandrov et al., 2013; This study	ftp://ftp.sanger.ac.uk/pub/cancer/AlexandrovEtAl/ Table S8
Coding sequence for wt targets	COSMIC, Bamford et al., 2004	http://cancer.sanger.ac.uk/cosmic
IARC P53 Database R18	Bouaoun et al., 2016	http://p53.iarc.fr/
TRACERx	Jamal-Hanjani et al., 2017, Abbosh et al., 2017	
COSMIC Resistance Mutations v83	COSMIC, Bamford et al., 2004	http://cancer.sanger.ac.uk/cosmic
COSMIC Mutation Data v83	Bamford et al., 2004	http://cancer.sanger.ac.uk/cosmic

**Software and Algorithms**		

Maestro version 9.8.016	Schrödinger	https://www.schrodinger.com/maestro
MOE 2015.1001	Chemical Computing Group	https://www.chemcomp.com/
Osprey version 2.2beta	Chen et al., 2009, Gainza et al., 2013	http://www.cs.duke.edu/donaldlab/osprey.php
AmberTools16	Case et al., 2016	http://ambermd.org/AmberTools16-get.html

### Contact for Reagent and Resource Sharing

Further information and requests for resources and reagents should be directed to and will be fulfilled by the Lead Contact Julian Blagg (Julian.blagg@icr.ac.uk).

### Data and Software Availability

Data are available upon request to the Lead Contact.

### Method Details

#### Structure Selection and Preparation

When available, crystal structures of wild type protein were used. Formation of the Lys-Glu salt bridge was the minimum requirement for kinase co-factor structures to be considered representative of an active conformation. Due to the paucity of high resolution ATP-bound kinase protein structures, we employed ADP or ATP analogue complexes for all kinase structures representative of endogenous co-factor binding. A list of employed PDB entries is provided in Table S7. All crystal structures were prepared using the Preparation wizard (Sastry et al., 2013) in Maestro version 9.8.016 (Schrödinger Release, 2014). Bond orders were assigned and hydrogens were added. Zero-order bonds to metals and disulphide bonds were created and selenomethionines were converted to methionines. Missing side chains were added and all waters beyond 5 Å from heteroatoms, buffer compounds, and additional chains were deleted. Ionization states were generated using Epik. H-bonds were assigned, water conformations were sampled and structures minimized using default settings.

Residues within 12 Å of the ligand were selected using the selector tool in MOE 2015.1001 (Molecular Operating Environment (MOE), 2017) and only selected residues were included in the input structure. As described in the Osprey documentation, the protonation state of histidines was defined in the pdb input file, all HETATM identifiers were replaced by ATOM, and chain information was deleted.

#### Evaluation of Ligand Affinity

Osprey version 2.2beta (Chen et al., 2009, Gainza et al., 2013), using the Amber94 force field, was employed to evaluate the impact of protein mutation on ligand binding. All non-peptide interaction partners were parameterized using Antechamber (AmberTools16 (Case et al., 2016)) as described in the Osprey documentation and only the selected crystal structure protein conformation was used. Small molecule ligands were allowed to rotate and translate during the calculations.

Residues within 5 Å of the ligand were systematically mutated to all other possible amino acids, except if they were known to be crucial for catalytic activity; namely the AspPheGly motif, catalytic Asp, and Lys-Glu salt bridge in kinases. All three His protonation states were included; His was only considered as a resistant mutation when all protonation states were predicted to confer resistance. Mutation to Pro is not supported by the Osprey package. In cases where ligands form specific water-mediated interactions with the protein, the water molecules were included and defined as co-factor as suggested in the Osprey documentation. Only one residue at a time was allowed to mutate and WT rotamers were included for all mutable residues. Residue positions were investigated either individually or in pairs; this led to multiple WT K* scores per protein-ligand complex and the average WT (avwt) score was used henceforth. The ratio of the endogenous ligand to drug K* score (= K* ratio) was calculated (Frey et al., 2010, Reeve et al., 2015) to evaluate the effect of each possible mutation on drug binding in relation to binding of the endogenous ligand. The log score of the K* ratio was subsequently used. The range of WT scores (range_wt_) across log-units was used to determine the variation range of the method for each input structure which results from the quality of the input model. (Figure S1). A decrease in drug affinity for a mutant compared to the WT protein has the potential to cause resistance, the activity cut-off for mutants was defined as a log K* ratio higher than the average WT protein value plus the inherent variation range of the method as described in Equation 1:

(Equation 1) $$cut-off=\log\left(K^{*}ratio_{avwt}\right)+range_{wt}$$

Every mutation retrieving a value higher than the cut-off was considered as conferring resistance.

The avwt score was used to define K* score values for residue positions that were only investigated for either endogenous ligand or drug. This was the case if a residue was within 5 Å of one but not the other.

#### Calculation of relP

The mutation type probabilities were taken from Alexandrov et al. (Alexandrov et al., 2013). The relevant signatures were extracted based on the cancer type for which the drug was approved. In detail, the signatures operating in stomach cancer and lung adenocarcinoma were used to calculate relative probabilities for KIT/ALK and EGFR small molecule drugs, respectively. Colorectal cancer signatures were employed for EGFR-antibody complexes. No signatures for CML (relevant for inhibitors of Abl) have been reported.

The original values of the signatures (x_s_) were normalized according to their total contribution to mutational load and the number of samples in which the signature could be detected (c_s_) (Figure S2). The normalized signature probabilities of all relevant signatures were added to give the overall probability for a specific base exchange in a particular cancer type (=SPM probability, pSPM) as described in Equation 2:

(Equation 2) $$pSPM=\;\sum_{s_{1}}^{s_{n}}c_{s}*x_{s}$$

where:

s_1_ and s_n_ are the first and n^th^ signature contributing to the mutational load in a particular cancer type.

The dataset of cancer-specific SPM probabilities is provided in Table S8 in the Supplemental Information.

To calculate the relP for a specific amino acid missense mutation, the coding sequence for each amino acid residue and their 5’ and 3’ neighbouring bases were extracted from the COSMIC database (Bamford et al., 2004). Signature-derived pSPMs (as described above) were used to define the relP for a specific amino acid exchange according to Equation 3:

(Equation 3) $$relP=\;\sum_{t_{1}}^{t_{n}}pSPM_{1}*pSPM_{2}$$

where:

t_1_ and t_n_ are the first and n^th^ combination of base exchanges resulting in the desired protein residue change.

pSPM_1_ and pSPM_2_ are the overall probability for a specific base exchange 1 and 2 in a particular cancer type. DPMs have been reported in cancer patients with similar frequency to insertions (Table S1) and were therefore included in the workflow. Please note, that only pSPM_1_ is required if a protein residue mutation can be facilitated by a single base exchange (SPM), whereas both pSPM_1_ and pSPM_2_ are used for a DPM.

Whenever multiple different mutations led to the same amino acid exchange, the individual SPM probabilities were added. The calculation of relP, exemplified on the EGFR T790M mutation in adenocarcinoma, is depicted in Figure S2.

### Quantification and Statistical Analysis

Microsoft Excel was used to calculate the mean ± SEM across all 18 target-interaction partner case studies (=n) as reported in the Discussion.

## Supporting Citations

The following references appear in the Supplemental Information: Garrett et al., 2002, Stamos et al., 2002, Mol et al., 2003, Mol et al., 2004, Li et al., 2005, Levinson et al., 2006, Tokarski et al., 2006, Yun et al., 2007, Cowan-Jacob et al., 2007, Gajiwala et al., 2009, Lee et al., 2010, Lu et al., 2010, Cui et al., 2011, Garai et al., 2012, Levinson and Boxer, 2012, Chaikuad et al., 2014, Friboulet et al., 2014, Sander et al., 2015, Menichincheri et al., 2016, Sickmier et al., 2016.

## Supplemental Information

Document S1. Figures S1 and S2 and Table S1

Table S2. Full List of Predicted ERK Resistance Mutations, Related to Table 1

Table S3. Full List of Predicted KIT Resistance Mutations, Related to Table 2

Table S4. Full List of Predicted EGFR Resistance Mutations, Related to Table 3

Table S5. Full List of Predicted Abl Resistance Mutations, Related to Table 4

Table S6. Full List of Predicted ALK Resistance Mutations, Related to Table 5

Table S7. PDB Entries of Evaluated Ligand-Target Complexes, Related to the STAR Methods

Table S8. Single Nucleotide Mutation Probabilities in Specific Cancer Types, Related to the STAR Methods

Document S2. Article plus Supplemental Information
