# Supplementary material for: Combining Mutational Signatures, Clonal Fitness, and Drug Affinity to Define Drug-Specific Resistance Mutations in Cancer
Source: Cell Chem Biol. 2018 Nov 15;25(11):1359–1371.e2. doi: 10.1016/j.chembiol.2018.07.013 (PMC6242700; doi:10.1016/j.chembiol.2018.07.013)
Supplement: Document S1. Figures S1 and S2 and Table S1 [file mmc1.pdf]

Cell Chemical Biology, Volume *25*

Supplemental Information

Combining Mutational Signatures, Clonal Fitness, and Drug Affinity to Define Drug-Specific Resistance Mutations in Cancer

Teresa Kaserer and Julian Blagg

Supplemental Information

Combining mutational signatures, clonal fitness and drug affinity to define drug-specific resistance mutations in cancer

Teresa Kaserer,* Julian Blagg*†

Cancer Research UK Cancer Therapeutics Unit, The Institute of Cancer Research, London, SM2 5NG, UK

* Corresponding authors: teresa.kaserer@icr.ac.uk, julian.blagg@icr.ac.uk

† Lead contact: julian.blagg@icr.ac.uk

Table S1. Proportion of DPMs and Insertions among unique cancer mutations. Related to STAR Methods, Introduction and Results.

Dataset	DPMs (%)	Insertions (%)	Comments	Reference
IARC P53 Database	4.5	12.1	Version R18	http://p53.iarc.fr/ (Bouaoun et al., 2016)
TRACERx	1.2	0.7	The whole cohort	(Jamal-Hanjani et al., 2017)
TRACERx	1.2	1.0	The relapse and autopsy cohort	(Abbosh et al., 2017)
COSMIC Resistance Mutations	5.3	1.5	Version 83 (access date 05/01/2018) Only mutations with defined genetic alteration.	http://cancer.sanger.ac.uk/cosmic (Bamford et al., 2004)
COSMIC Mutation Data	4.1	7.6	Version 83 (access date 05/01/2018)	http://cancer.sanger.ac.uk/cosmic (Bamford et al., 2004)

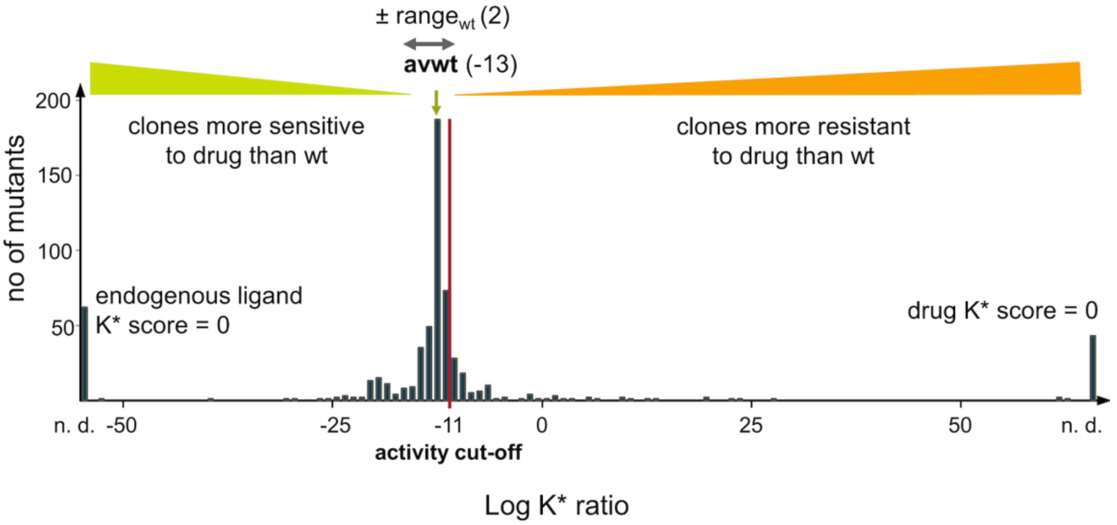

Figure S1. Log K* ratio distribution exemplified for the drug-target pair SCH772489-ERK2. Data related to Discussion and STAR Methods. The number of mutants with a similar K* ratio is plotted against the binned log K* ratio value. Mutants with a score lower than average wt (avwt) – range$_{wt}$ indicate increased drug binding (=sensitivity) until the case where a mutant can still interact with the drug, but not the endogenous ligand (endogenous ligand K* score = 0). Scores higher than the avwt + range$_{wt}$ indicate mutants with increasing resistance to drug, until the case where drug binding is completely abrogated (drug K* score = 0). In the SCH772489-ERK2 example, the logK* score of the avwt is -13 and the wt values span across 2 log-units (= range$_{wt}$). The cut-off was therefore defined as -11. n. d. not defined, when a K* score of 0 was retrieved, the log value could not be defined. The Figure was generated with DataWarrior (Sander et al., 2015).

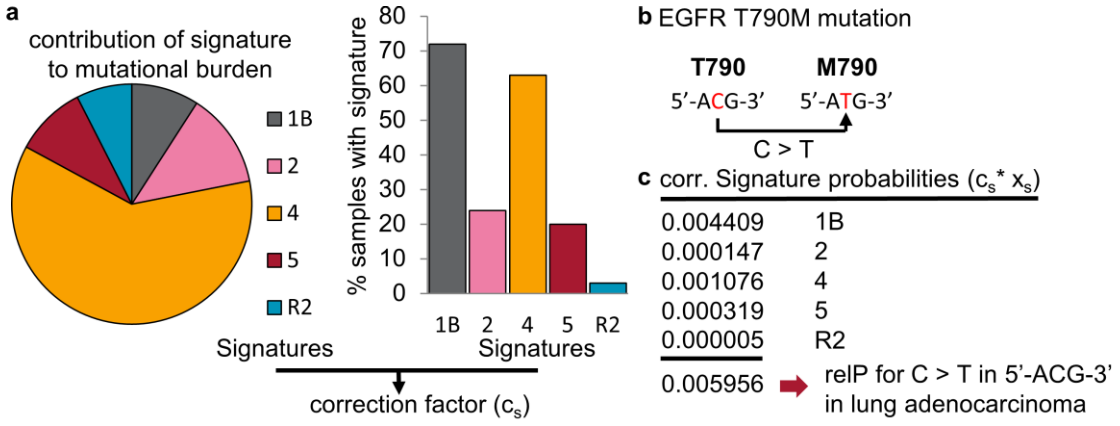

Figure S2. Calculation of relPs exemplified for EGFR T790M in lung adenocarcinoma. Data related to Star Methods. (**a**) Both the contribution of a signature to the mutational burden observed in a particular cancer type, lung adenocarcinoma in this example, and the percentage of patient samples in which the signature was found to be operating, were used to calculate a correction factor (c_S). Signature probabilities reported by Alexandrov et al. (Alexandrov et al., 2013) were multiplied by c_S to normalize signatures for their contribution to lung adenocarcinoma (c_S*x_S). For example, Signature 1B contributes 9.1% of mutations observed in lung adenocarcinoma and was detected in 72% of cancer samples. The c_{1B} for Signature 1B (0.091 x 0.72 = 0.06552) in the context of lung adenocarcinoma was used to normalize the original Signature 1B probabilities (x_B) (Alexandrov et al., 2013). Besides Signature 1B, Signatures 2, 4, 5, and R2 were also reported to contribute to the mutation spectrum in lung adenocarcinoma. Taken together the corrected signature probabilities reflect the likelihood that a particular mutation will occur in that cancer type (pSPM). (**b**) The EGFR T790M mutation is generated by a C > T base change in the context of a 5'-ACG-3' sequence. (**c**) The corrected (corr.) signature probabilities (c_S*x_S) for C > T mutation in the 5'-ACG-3' context for all relevant mutational signatures were added to give the relP for the T790M mutation in lung adenocarcinoma.

Please note, that in this case pSMP = relP, because there is only one base change which can facilitate the T790M mutation. Figure S2a was adapted with permission from Springer Nature: Nature (Alexandrov et al., 2013), COPYRIGHT 2013.
